# The immunoecology of occult hepatitis B virus infection: genetic remodeling and the immune-mediated microcosm

**DOI:** 10.3389/fimmu.2026.1899891

**Published:** 2026-07-27

**Authors:** Feng Wang, Le Wang, Zhiguo Xu, Zequn Ou, Yun Wang, Haiying Yang, Jingxian Fei, Jingxian Song, Yizhu Chen, Ke Lv

**Affiliations:** 1 Huzhou Central Blood Station, Huzhou, China; 2 Huzhou College of Life and Health, Huzhou, China; 3 Union East China Stem Cell Genetic Engineering Co., Ltd., Huzhou, China

**Keywords:** epigenetic regulation, genetic remodeling, hepatocellular carcinoma, immune surveillance, immune-mediated microcosm, occult hepatitis B virus infection, precision medicine

## Abstract

Occult hepatitis B infection (OBI) is characterized by the persistence of replication-competent hepatitis B virus (HBV) DNA in the absence of detectable hepatitis B surface antigen (HBsAg). Although typically clinically silent, OBI remains associated with transfusion-transmitted infection, viral reactivation during immunosuppression, and an increased risk of hepatocellular carcinoma (HCC). The mechanisms underlying this persistent low-replicative state remain incompletely understood. In this review, we summarize current evidence supporting the concept that OBI is maintained through dynamic interactions among viral genetic adaptation, host genetic and epigenetic regulation, and the intrahepatic immune microenvironment. Building upon these observations, we propose the immune-mediated microcosm as a conceptual framework describing how these processes may interact to sustain long-term immune equilibrium. We further discuss emerging evidence for coordinated viral and host genetic remodeling, immunometabolic regulation, and immune checkpoint signaling, while explicitly distinguishing mechanisms supported by direct evidence in OBI from those inferred from chronic hepatitis B, hepatocellular carcinoma, or experimental models. Finally, we examine the implications of this framework for biomarker discovery, risk stratification, and therapeutic development, while emphasizing the current evidential limitations and the need for direct validation in human OBI. Rather than providing a definitive mechanistic model, this framework is intended to facilitate mechanistic investigation, generate experimentally testable hypotheses, and help identify priorities for future research into OBI pathogenesis.

## 1 Introduction

Occult hepatitis B virus infection (OBI) is characterized by the persistence of replication-competent hepatitis B virus (HBV) DNA in the liver and/or blood despite negative hepatitis B surface antigen (HBsAg) testing (1). Traditionally regarded as either a resolved infection or a limitation of serological detection, OBI is now recognized as a biologically distinct state of long-term host–virus persistence rather than a transient diagnostic phenomenon (1, 2). This distinction has important clinical implications. Because routine blood donor screening relies primarily on HBsAg negativity, OBI remains the principal source of residual transfusion-transmitted HBV worldwide (2, 3). Although individual-donation nucleic acid testing has substantially improved blood safety, intermittent low-level viremia may still remain below current detection thresholds, allowing a small but persistent risk of transmission (4, 5).

The clinical significance of OBI extends well beyond transfusion medicine. Its prevalence broadly parallels regional HBV endemicity and remains substantial in both the general population and blood donor cohorts (6, 7). Persistent occult infection also represents an important reservoir with implications for HBV elimination efforts, particularly in regions with a high background prevalence of infection (6, 8). In addition, OBI is increasingly recognized as a clinically relevant condition because it predisposes susceptible individuals to HBV reactivation during immunosuppressive therapy and is independently associated with hepatocellular carcinoma, including non-cirrhotic disease (9–12).

Despite these well-established clinical associations, the biological mechanisms that enable OBI to persist for decades remain incompletely understood. Current evidence suggests that viral persistence cannot be explained solely by reduced viral replication or immune escape. Rather, OBI appears to arise from dynamic interactions among viral genetic adaptation, host regulatory mechanisms, and the intrahepatic immune environment. Viral surface antigen variants may reduce antigen detectability, whereas transcriptionally restrained covalently closed circular DNA (cccDNA) provides a durable template for persistence. At the same time, inherited variation in host immune regulation, including HLA class II polymorphisms associated with OBI susceptibility, indicates that host genetic factors contribute substantially to long-term immune control (13–15).

How this state of persistence is maintained remains one of the central unresolved questions in HBV biology. Existing models largely attribute OBI to reduced viral replication, impaired antigen detection, or isolated immune escape mechanisms. Although each contributes to viral persistence, none adequately explains how viral replication, immune surveillance, and tissue integrity remain balanced over decades without progression to overt hepatitis in most individuals.

To address this gap, we propose the concept of an immune-mediated microcosm as a framework for understanding OBI. Rather than referring simply to the hepatic immune microenvironment, this framework describes a coordinated local regulatory network in which viral adaptation, host immune regulation, and intrahepatic tissue homeostasis interact to maintain a dynamic equilibrium. Within this network, antiviral immunity is neither fully activated nor completely silenced. Instead, residual immune surveillance is preserved while excessive inflammatory injury is restrained, allowing persistent viral control without complete viral elimination. We refer to this adaptive equilibrium as armed coexistence.

This framework differs conceptually from both immune tolerance and functional cure. Unlike immune tolerance, it assumes that antiviral effector function is retained but is continuously modulated according to the exceptionally low antigen burden characteristic of OBI. Unlike functional cure, viral persistence remains biologically active through transcriptionally constrained but replication-competent cccDNA. The resulting equilibrium is therefore viewed as an actively regulated state rather than passive coexistence.

Central to this concept is the hypothesis that antigen quantity functions not merely as a marker of viral replication but also as a determinant of immune behavior. Under conditions of sustained high antigen exposure, as observed in chronic hepatitis B, persistent T-cell receptor stimulation promotes progressive functional exhaustion. By contrast, the markedly reduced antigen burden in OBI may establish a distinct immunological set-point in which inhibitory pathways constrain excessive immune activation while preserving the capacity for immune responsiveness. This hypothesis provides a mechanistic framework linking viral persistence to local immune regulation and forms the conceptual basis for the analyses presented throughout this Review.

An important implication of this framework is that the biological behavior of OBI may be determined less by the absolute presence of HBV than by the quantity and spatial distribution of viral antigen within the liver. We therefore propose that OBI is maintained within an antigen threshold compatible with effective immune surveillance but insufficient to trigger sustained immunopathology. Below this range, immune surveillance may progressively diminish, whereas persistent increases in antigen burden may promote immune activation, viral reactivation, or inflammatory remodeling. The molecular determinants governing these transitions remain incompletely understood and require direct investigation in human OBI cohorts.

**Figure 1:**
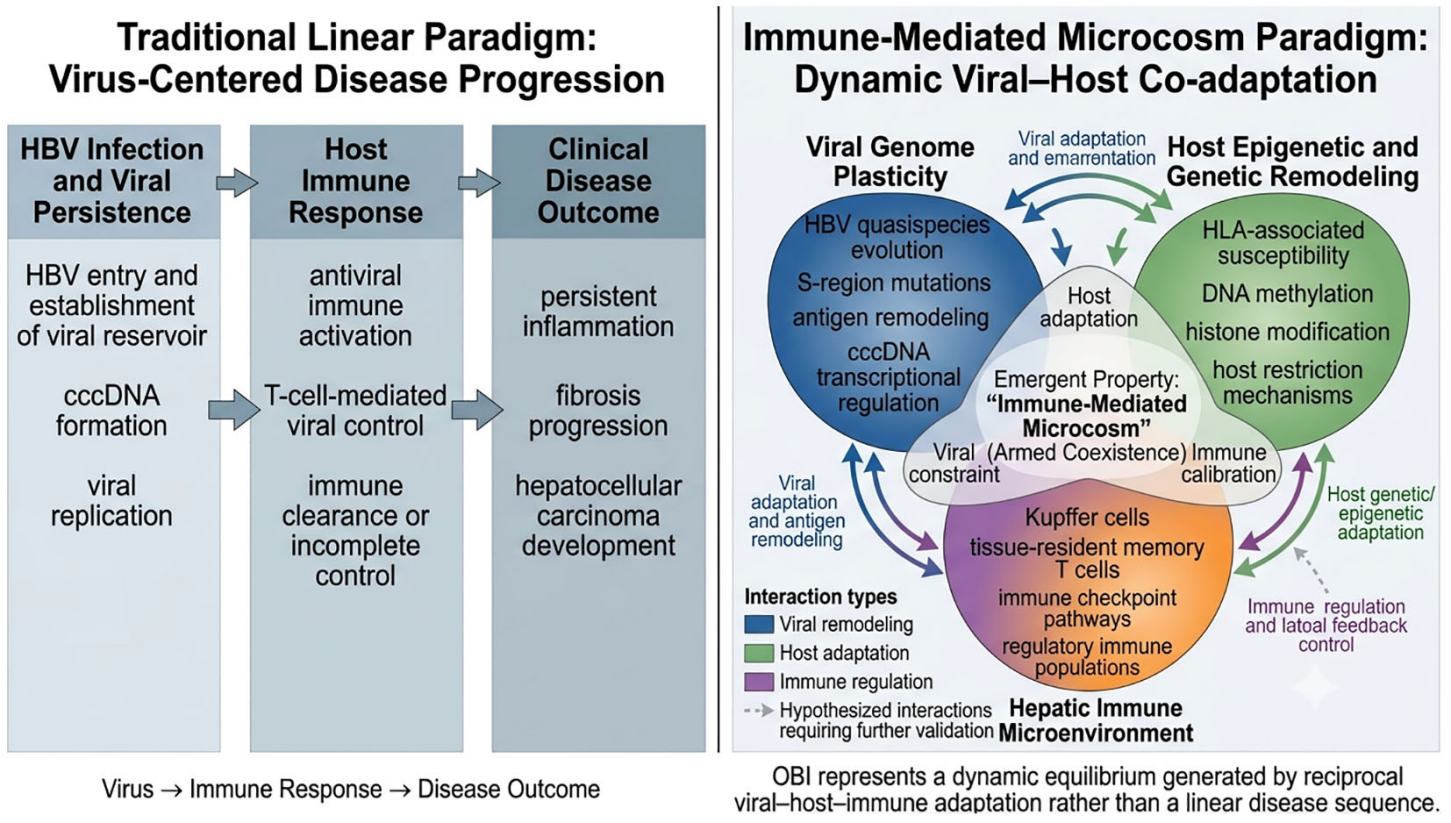
Paradigm shift in occult HBV infection research: from a linear virus-centered model to a dynamic immune-mediated microcosm framework. The left panel illustrates the traditional linear paradigm, in which HBV infection is interpreted as a sequential process linking viral persistence, host immune responses, and clinical disease outcomes. Three functional modules represent viral reservoir establishment, antiviral immune responses, and downstream pathological consequences, connected by unidirectional progression arrows. The right panel presents the immune-mediated microcosm framework proposed in this Review. OBI is conceptualized as a dynamic equilibrium generated by reciprocal interactions among three adaptive domains: viral genome plasticity, host genetic and epigenetic remodeling, and the hepatic immune microenvironment. Rather than representing homeostasis as an independent regulatory component, the immune-mediated microcosm is depicted as an emergent property arising from continuous interactions among these systems. Colored arrows indicate distinct interaction categories: blue represents viral remodeling and antigen adaptation, green represents host genetic and epigenetic adaptation, and purple represents immune regulation and feedback control. Dashed gray arrows denote hypothesized interactions requiring further experimental validation. Together, these interconnected processes illustrate OBI as a state of reciprocal viral–host–immune adaptation rather than a unidirectional disease sequence.

Building upon this perspective, the present Review integrates recent advances in viral genetics, epigenetic regulation, immunometabolism, and intrahepatic immune organization to examine how reciprocal interactions between HBV and the host may sustain long-term occult infection. We further discuss how disruption of this equilibrium may contribute to HBV reactivation and hepatocarcinogenesis, evaluate the current strength and limitations of the available evidence, and propose experimentally testable hypotheses together with candidate operational parameters for future validation. Rather than presenting OBI as a static virological state, this Review considers it a dynamic host–virus ecosystem whose behavior emerges from coordinated interactions across multiple biological scales (**Figure 1**).

## 2 Drivers of genetic remodeling: viral evolution and host rewriting

The persistence of OBI involves continuous, bidirectional genetic remodeling under reciprocal selective constraints. On the viral side, adaptive mutations and post-translational modifications enable immune evasion. On the host side, HLA polymorphisms, DNA methylation, histone modifications, and metabolite-derived acylations alter the transcriptional template.

### 2.1 Viral quasispecies optimization under immune pressure

A major limitation of current evidence is that most studies on OBI-associated viral mutations utilize cross-sectional cohorts. Consequently, whether the mutation patterns observed in these cohorts reflect the long-term evolutionary trajectory of the virus within individual hosts remains to be systematically examined.

In OBI, HBV faces a trade-off between sustaining quasispecies persistence and balancing replication fidelity against antigen expression. S-region mutations represent a well-documented adaptation. Screening of 91,037 patients identified immune-escape mutations in 59.68% of OBI cases, with sK122R impairing HBsAg secretion (16). PreS/S profiling in northern Chinese blood donors confirmed that MHR amino acid replacements and preS deletions are common molecular signatures (17). Functional validation has since shown that sT5A and sF20S reduce HBsAg output (18).

Quasispecies composition is inherently dynamic. Under immunosuppression, low-abundance variants can be positively selected to achieve dominance. Svicher et al. reported that 75.9% of patients with HBV reactivation harbored fixed HBsAg escape mutations at a median intra-host prevalence of 73.3% (19). Ultra-deep sequencing further revealed that dominant pre-S2 mutant quasispecies are more prevalent in OBI-related HCC than in HBsAg-positive HCC, suggesting that quasispecies composition may differentially influence oncogenic potential (20). Similar remodeling occurs during prolonged antiviral drug exposure, which drives the accumulation of HBsAg stop codons and escape mutations (21). Notably, pre-existing drug-resistance variants are detectable even in treatment-naïve populations (22), reflecting substantial baseline viral diversity rather than direct evidence of immune escape. Whether these variants contribute to immune evasion in OBI remains unknown.

Beyond canonical mutations, the virus can modify its surface antigen via post-translational glycosylation. Additional N-linked sites within the “a” determinant can sterically shield antibody epitopes, yielding persistently negative HBsAg results (23, 24). Substitutions such as T123N and K160N exemplify this escape route (25). In hemodialysis patients subjected to repeated immunologic pressure, these mutations accumulate within the “a” determinant (26). These observations suggest that the OBI quasispecies does not merely undergo passive decay but is subject to active optimization driven by immune selection.

### 2.2 HLA polymorphism and immune surveillance boundaries

Host MHC molecules, particularly HLA class II, dictate the capacity to present viral epitopes to CD4+ T cells, thereby defining the boundaries of immune surveillance. A multicenter whole-exome study identified four HLA SNPs associated with low OBI susceptibility and proposed a dual-mechanism model in which HLA class I variants impair cytotoxic responses, whereas HLA class II variants disrupt humoral immunity (14). Haplotype analyses identified HLA-DRB1*07:01-DQB1*02:02 (OR = 3.489) and HLA-DRB1*09:01-DQB1*03:03 (OR = 2.370) as key susceptibility markers (15). High-resolution genotyping subsequently revealed shared amino acid signatures in the DRβ1 chain associated with OBI, providing structural clarity to this antigen presentation deficit (27). Independent validation at the HLA-DP locus confirmed that carriers of the rs3077 minor allele face an elevated risk of OBI (OR = 3.87) (28). The contribution of HLA class II polymorphisms to HBV infection outcomes is further supported by genotyping-based association studies in chronic HBV (29), and multiethnic replication indicates that HLA-DP and HLA-DQ variants influence HBV clearance and chronicity across diverse populations (30). Southeast Asian cohorts have likewise revealed distinct HLA alleles associated with infection chronicity or resolution (31), consistent with the population-specific architecture of HLA-conferred susceptibility.

One caveat deserves emphasis: these genetic associations define statistical risk rather than a deterministic fate. An individual carrying a high-risk haplotype may still maintain long-term OBI control if other components of the immune microcosm compensate. Although this compensatory mechanism remains to be systematically examined, we summarize the key HLA-OBI associations in **Table 1**.

**Table 1:** Key host HLA alleles/haplotypes associated with OBI susceptibility.

HLA allele/haplotype	Population	Association	Effect size (OR)	95% CI	Multiple testing/stratification control	Proposed immunological mechanism	Reference
HLA-DRB1*07:01-DQB1*02:02	Chinese Han	Susceptibility	3.489	1.82–6.68	Bonferroni correction for haplotype-wise comparisons; PCA for population stratification	Impaired CD4+ T-cell-dependent humoral immunity	(15)
HLA-DRB1*09:01-DQB1*03:03	Chinese Han	Susceptibility	2.370	1.28–4.39	Same as above	Defective antigen presentation, reduced peptide affinity	(15)
HLA-B*44:03-C*07:01G	Chinese Han	Susceptibility	—	—	Nominal P values reported; no formal multiple testing correction	Weakened HLA class I-restricted CTL response	(57)
HLA-DPA1 rs3077 (A allele)	Indonesian	Susceptibility	3.87	1.83–8.18	Logistic regression adjusted for age and sex; no correction for multiple SNPs	Altered Th1/Th2 polarization, decreased IFN-γ production	(28)
Shared DRβ1 amino acid signatures (DRB1*07:01, *09:01, *04:03, etc.)	Chinese Han	Susceptibility	—	—	Sequence-based clustering; not applicable	Amino acid substitutions in the antigen-binding groove impair peptide presentation	(27)
HLA-DP/DQ region SNPs (rs2856718, rs9277535, etc.)	Chinese Han	Protective	—	—	Genome-wide significance threshold (P < 5×10^−8^); PCA for population stratification	Enhanced antigen presentation, robust humoral response	(14)

Only studies that directly phenotyped OBI and reported statistically significant associations after multivariable adjustment are included. OR, odds ratio; CI, confidence interval. “—” indicates the OR or CI was not reported or not applicable (e.g., for amino acid signatures). Multiple testing and population stratification control methods are listed as described in the original publications; where not explicitly stated, “not reported” is noted. Detailed statistical parameters should be verified against the original references.

### 2.3 Epigenetic silencing and de-repression of cccDNA

The transcriptional activity of cccDNA is tightly regulated by host epigenetic mechanisms. Existing as an episomal minichromosome within the hepatocyte nucleus, the precise degree of residual transcription from cccDNA in OBI remains highly contested.

CpG island methylation suppresses viral promoter activity, inversely correlating with viral replication levels (32–34). Genome-wide mapping has revealed a distinct chromatin organization in which H3K9me3 and H3K27me3 enrichment coincides with transcriptional quiescence (35). SIRT3 and SIRT7 catalyze H3K9 deacetylation and H3K122 desuccinylation, respectively, cooperating with SUV39H1/SETDB1 to maintain cccDNA in a repressed state (36, 37). Conversely, the host factor NQO1 stabilizes HBx; pharmacological NQO1 inhibition using dicoumarol promotes HBx degradation, thereby reducing cccDNA transcription (38). On the viral side, HBx interacts with the DDB1-CUL4 ubiquitin ligase to target Smc5/6 for degradation and antagonizes SETDB1-mediated H3K9me3 deposition, thereby relieving the chromatin-mediated barrier to transcription (39, 40).

Whether cccDNA silencing in OBI is ever absolute remains an unresolved question, with evidence supporting both perspectives. The case for profound silencing rests on the hypermethylation of cccDNA CpG islands (32, 33), the enrichment of repressive histone marks in low-replicative states (35), and the frequent failure of conventional assays to detect intrahepatic pgRNA in OBI (41). However, recent single-cell data from patients who achieved functional cure (defined by HBsAg seroclearance) reveal persistent, low-level cccDNA and pgRNA (42). This finding indicates that transcriptional quiescence may not be absolute even in the most clinically favorable scenarios. Similarly, in cryptogenic HCC patients with OBI, intrahepatic pgRNA was detected in approximately 52% of cases at a median of roughly 0.0001 copies per cell; furthermore, carriers with detectable serum HBV DNA were more likely to harbor quantifiable intrahepatic pgRNA (41). These data suggest a clinical spectrum: some individuals maintain near-silent cccDNA, whereas others exhibit low-grade transcriptional leak. Such leak, though insufficient to produce detectable HBsAg, could sustain basal viral fitness and drive the accumulation of HBV DNA integrations over time.

Resolving this controversy is hampered by methodological constraints intrinsic to studying low-copy, focally distributed viral genomes. Even droplet digital PCR detects intrahepatic cccDNA in only about 52% of anti-HBc-positive OBI cases, at a median of 13 copies per 10^5^ cells, leaving the remainder below the limit of detection (43). Three recognized technical obstacles collectively reduce assay sensitivity in this low-copy setting: high sequence homology to other HBV DNA forms, extremely low copy numbers per infected cell, and resistance to heat denaturation (44). Spatial heterogeneity further compounds the issue, as duplicate biopsies from the same liver show substantial variation in the proportion of HBV-positive hepatocytes (45). Consequently, sampling error increases significantly in specimens shorter than 5 mm or those containing fewer than four portal tracts (45, 46). Because residual cccDNA-bearing hepatocytes in OBI exist as scattered, isolated clones (47), a single negative biopsy cannot be interpreted as definitive evidence of viral clearance. Moreover, reliance on core liver biopsies inherently limits longitudinal studies in asymptomatic OBI carriers (48).

Fine-needle aspiration (FNA) has emerged as a less invasive alternative, with paired validation showing no significant differences in cccDNA quantification compared with core biopsies (49). However, the lower cellular yield of FNA raises questions about its suitability for chromatin immunoprecipitation (ChIP)-based techniques required to map histone modifications on low-abundance cccDNA minichromosomes. Current cccDNA ChIP-Seq protocols were validated primarily in *de novo*-infected HepG2 cells and high-viremia liver tissues (35); thus, their applicability to the sparse cccDNA pool in OBI remains to be established, as the number of molecules per reaction often falls below reliable enrichment thresholds. Emerging single-hepatocyte-resolved platforms may offer a solution. The recently developed scID-PCR method quantifies HBV-positive cells with single-cell resolution and has detected residual infection in patients with undetectable serum HBV DNA following antiviral therapy (50). Nonetheless, the transition from proof-of-principle to routine application in OBI remains considerable.

Several findings discussed in this section derive from direct OBI evidence (Tier 1): the detection of hypermethylated cccDNA CpG islands in OBI liver tissue (32, 33), the enrichment of repressive histone marks in low-replicative states (35), and the frequent absence of detectable intrahepatic pgRNA in OBI carriers (41, 51). The single-cell data demonstrating persistent low-level cccDNA and pgRNA in functionally cured patients (42) represent Tier 2 evidence—obtained from individuals who have achieved HBsAg seroclearance, a state that shares key immunological features with OBI but may not be identical. The functional studies of SIRT3, SIRT7, and HBx-mediated cccDNA regulation (36, 37, 39, 40) derive predominantly from HBV replication models and chronic hepatitis B liver tissue (Tier 2/Tier 3); their quantitative relevance to the residual cccDNA pool in OBI requires further validation.

### 2.4 Metabolite-driven post-translational modifications: lactylation and succinylation

One frontier that has attracted considerable interest concerns the potential role of metabolite-driven post-translational modifications in regulating the residual cccDNA pool. This frontier, however, remains largely unexamined in the context of OBI.Metabolic intermediates such as lactate and succinyl-CoA can covalently modify histones and non-histone proteins, thereby coupling cellular metabolic status to gene expression (52). The discovery of histone lactylation provided evidence that glycolytic lactate can influence chromatin activation (53), and H3K122 succinylation on cccDNA has been identified as a transcriptional activation mark reversed by SIRT7-catalyzed desuccinylation (36). In HBV-related HCC, HBx-mediated HK2 upregulation is associated with increased aerobic glycolysis and altered histone lactylation patterns (54).

An important question is whether HBx is barely detectable in OBI, why discuss HBx-driven lactylation at all? The honest answer is that the relevance of these modifications to OBI remains a hypothesis. We include this brief discussion not because the evidence is strong. It is not. Rather, we include it because the hypothesis is testable and worth articulating. Three specific questions define the current knowledge gap. First, whether HBx is expressed at sufficient levels in the low-replication OBI state to upregulate HK2 is unknown. Second, even if HK2 upregulation occurs, whether the resultant lactate accumulation reaches the millimolar-range threshold required for histone lactyltransferase activity is uncertain. Third, whether succinyl-CoA concentrations in OBI hepatocytes permit H3K122 succinylation at the cccDNA minichromosome has not been measured. In OBI carriers lacking significant hepatic inflammation, the Warburg effect may be minimally operative, and substrate concentrations for these modifications may be considerably lower than those in HCC or acute liver failure.

**Figure 2:**
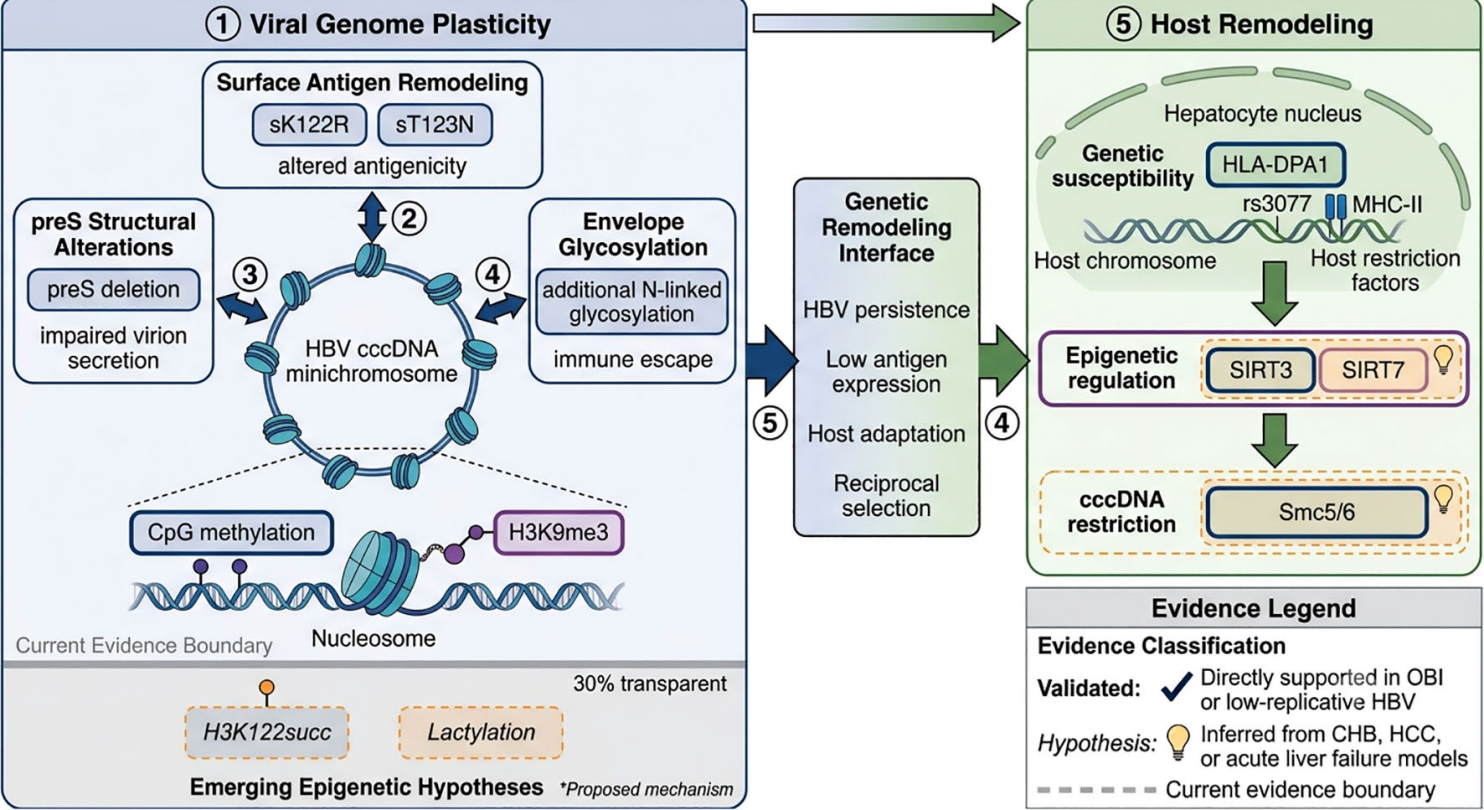
Reciprocal viral and host genetic remodeling underlying persistent occult HBV infection. The figure illustrates a conceptual framework describing reciprocal genetic remodeling between HBV and host factors during the establishment and maintenance of occult hepatitis B virus infection (OBI). The numbered workflow (①–⑤) summarizes the proposed interaction between viral genome plasticity, the genetic remodeling interface, and host adaptation. Viral remodeling (①–④) includes surface antigen alterations, preS structural changes, and envelope glycosylation patterns that may reduce antigen visibility, alter viral fitness, and facilitate persistence. These adaptive processes converge on the HBV cccDNA minichromosome, where epigenetic regulation contributes to transcriptional control. CpG methylation and H3K9me3 are supported by direct or low-replicative HBV evidence, whereas H3K122 succinylation and histone lactylation are shown as emerging hypotheses primarily inferred from chronic hepatitis B, hepatocellular carcinoma, or acute liver injury models. The central genetic remodeling interface represents a proposed zone of reciprocal adaptation linking persistent viral low-level antigen expression with host regulatory responses. Host remodeling (⑤) includes genetic susceptibility factors such as HLA-DPA1 variation, epigenetic regulators including SIRT3 and SIRT7, and intrinsic cccDNA restriction mechanisms involving Smc5/6.Evidence levels are indicated by visual classification: solid blue-bordered elements represent mechanisms supported by OBI or low-replicative HBV studies, whereas dashed orange-bordered elements denote proposed mechanisms requiring validation in human OBI samples. The current evidence boundary indicates unresolved biological questions. This model represents a hypothesis-generating framework of viral–host adaptation rather than a definitive linear mechanism.

The principal value of discussing these modifications may lie in the methodological challenge they pose. Whether technologies capable of detecting site-specific histone modifications on the extremely low-abundance cccDNA pool in OBI liver biopsies can be developed remains an open question. Emerging single-hepatocyte-resolved platforms, such as the recently reported scID-PCR method (50), represent early steps in this direction. The data on histone lactylation and succinylation derive almost entirely from HCC tumor specimens, HCC cell lines, and acute liver failure samples (Tier 3) (36, 53–56); their applicability to the residual cccDNA minichromosome in HBsAg-negative, low-copy OBI liver tissue has not been established, and the discussion in this section should be read as hypothesis-generating. **Figure 2** provides a schematic overview of the genetic remodeling events discussed in Section 2.

## 3 The hepatic immune-mediated microcosm: order and constraint

Long-term persistence of occult hepatitis B virus infection (OBI) depends not only on coordinated viral and host genetic remodeling but also on a locally regulated immune ecosystem that operates under conditions of extremely low antigen exposure. Rather than representing immune quiescence, this state reflects armed coexistence, in which antiviral surveillance is preserved while excessive immune activation is actively restrained. Within this framework, Kupffer cells, tissue-resident lymphocytes, immune checkpoint pathways, and the local metabolic milieu collectively maintain a dynamic but stable intrahepatic equilibrium.

### 3.1 From acute infection to occult equilibrium

A major obstacle to understanding OBI is the absence of longitudinal human studies capturing the transition from acute HBV infection to occult persistence. Consequently, current models are derived primarily from animal studies together with cross-sectional analyses of human liver tissue.

The proposed immune-mediated microcosm is thought to emerge during resolution of acute infection. Acute HBV is characterized by vigorous intrahepatic CD8^+^ T-cell responses, whereas prolonged antigen exposure promotes progressive expression of inhibitory molecules including PD-1 and TOX (58, 59). When viral clearance remains incomplete, sustained immune pressure reduces the residual cccDNA pool to transcriptionally constrained levels while preserving replication competence (32–34). The resulting HBsAg-negative state permits long-term persistence of hepatocytes carrying residual cccDNA and integrated HBV DNA without overt hepatic inflammation (41, 60, 61). Rather than reflecting immune failure, this equilibrium is proposed to arise from coordinated regulation in which antiviral effector activity and immunoregulatory mechanisms remain simultaneously engaged.

### 3.2 Cellular composition and metabolic constraints

Although the regulation of intrahepatic CD8^+^ T-cell function has been extensively investigated in experimental systems, its precise role in human OBI remains incompletely defined. Intravital imaging demonstrates that effector CD8^+^ T cells patrol hepatic sinusoids and engage infected hepatocytes in a non-migratory manner (62). Within this network, the KC2 subset of Kupffer cells restores CD8^+^ T-cell function through IL-2 secretion, whereas HBV-specific CD4^+^ T cells license Kupffer cells to produce IL-27, thereby limiting the development of T-cell dysfunction (63, 64). These mechanisms are supported primarily by HBV transgenic and human liver chimeric mouse models (Tier 3) and remain to be confirmed in human OBI. Under the low-antigen conditions characteristic of OBI, tissue-resident memory T (TRM) cells constitute the predominant intrahepatic effector population. Human liver studies demonstrate their long-term persistence and rapid recall responses following antigen re-exposure (65) (Tier 1).

Antiviral surveillance is balanced by multiple immunoregulatory populations. Myeloid-derived suppressor cells (MDSCs) deplete extracellular L-arginine through arginase activity and secrete IL-10 and TGF-β, thereby promoting regulatory T-cell differentiation (66, 67). IL-10-producing regulatory B (Breg) cells further suppress IFN-γ and TNF-α production by HBV-specific CD8^+^ T cells through IL-10-dependent mechanisms (68). Evidence supporting the roles of MDSCs, Treg cells, and Breg cells in OBI is derived predominantly from chronic HBV cohorts (Tier 2), and their quantitative contribution to armed coexistence in human OBI remains to be established.

The metabolic microenvironment provides an additional layer of regulation. By controlling the availability of arginine, tryptophan, and cysteine, hepatocytes influence the balance between CD8^+^ T-cell activation and functional restraint (69, 70). A recent multi-omics study identified a peripheral immunometabolic signature that distinguishes OBI from HBsAg-positive infection (71). Whether these peripheral signatures accurately reflect intrahepatic immune metabolism remains uncertain. Among the pathways implicated, activation of the tryptophan–kynurenine axis through increased indoleamine 2,3-dioxygenase (IDO) activity promotes kynurenine accumulation, AhR signaling, suppression of T-cell effector function, and expansion of regulatory T cells (72). Current evidence for this pathway in OBI remains indirect and is inferred largely from chronic HBV studies (Tier 2).

### 3.3 Inhibitory checkpoint and cytokine networks

The PD-1/PD-L1 and Tim-3/Galectin-9 pathways constitute the principal inhibitory checkpoint networks within the hepatic immune-mediated microcosm. PD-L1 expression on monocyte-derived cells is enriched in inactive HBV carriers, whereas Gal-9^+^ monocytes predominate during the immune-tolerant phase, suggesting stage-dependent regulation of checkpoint activity (58, 73). In low-replicative HBV states, intrahepatic accumulation of PD-1^+^CD8^+^ T cells has been consistently observed (58) (Tier 2). Importantly, sustained antigen exposure may induce multiple dysfunctional T-cell states, including antigen-adapted hyporesponsiveness that is mechanistically distinct from classical terminal exhaustion (59).

Innate immune regulation follows a similar pattern. A CX3CR1^+^KLRC2^−^CD16^hi^ NK-cell subset capable of antibody-dependent cellular cytotoxicity (ADCC) is associated with improved viral control (74), and adaptive NKG2C^+^ NK cells exhibit antiviral activity in preclinical HBV models (75). Conversely, Kupffer cell-derived IL-10 together with TGF-β establishes a local inhibitory cytokine environment that limits excessive immune activation through the CD163–IL-10 axis (76). These regulatory mechanisms are supported primarily by studies in chronic HBV infection and experimental systems (Tier 2/Tier 3), and their contribution to immune regulation in human OBI remains to be determined.

### 3.4 Metabolic–immune plasticity

Whether immune dysfunction in OBI can be therapeutically reversed without disrupting hepatic immune homeostasis remains an important unresolved question.

HBV-specific CD8^+^ T cells exhibiting functional impairment display mitochondrial dysfunction characterized by increased reactive oxygen species production and membrane depolarization, both of which are reversible following metabolic restoration (69). Within the same individual, metabolically flexible HBV-specific CD8^+^ T-cell subsets retain antiviral function, whereas dysfunctional subsets progressively lose glycolytic capacity and become increasingly dependent on oxidative phosphorylation (70). These observations support the concept that metabolic interventions targeting pathways such as acetyl-CoA metabolism may partially restore antiviral function through epigenetic reprogramming (77). Consistent with this possibility, HBV-specific CD4^+^ cytotoxic T lymphocytes identified in chronically infected livers have been associated with inflammation resolution and normalization of alanine aminotransferase levels (71). Collectively, these findings indicate that T-cell dysfunction is not necessarily irreversible, although whether comparable metabolic reprogramming can safely restore immune function in the low-antigen environment characteristic of OBI remains unknown.

### 3.5 Model limitations

**Figure 3:**
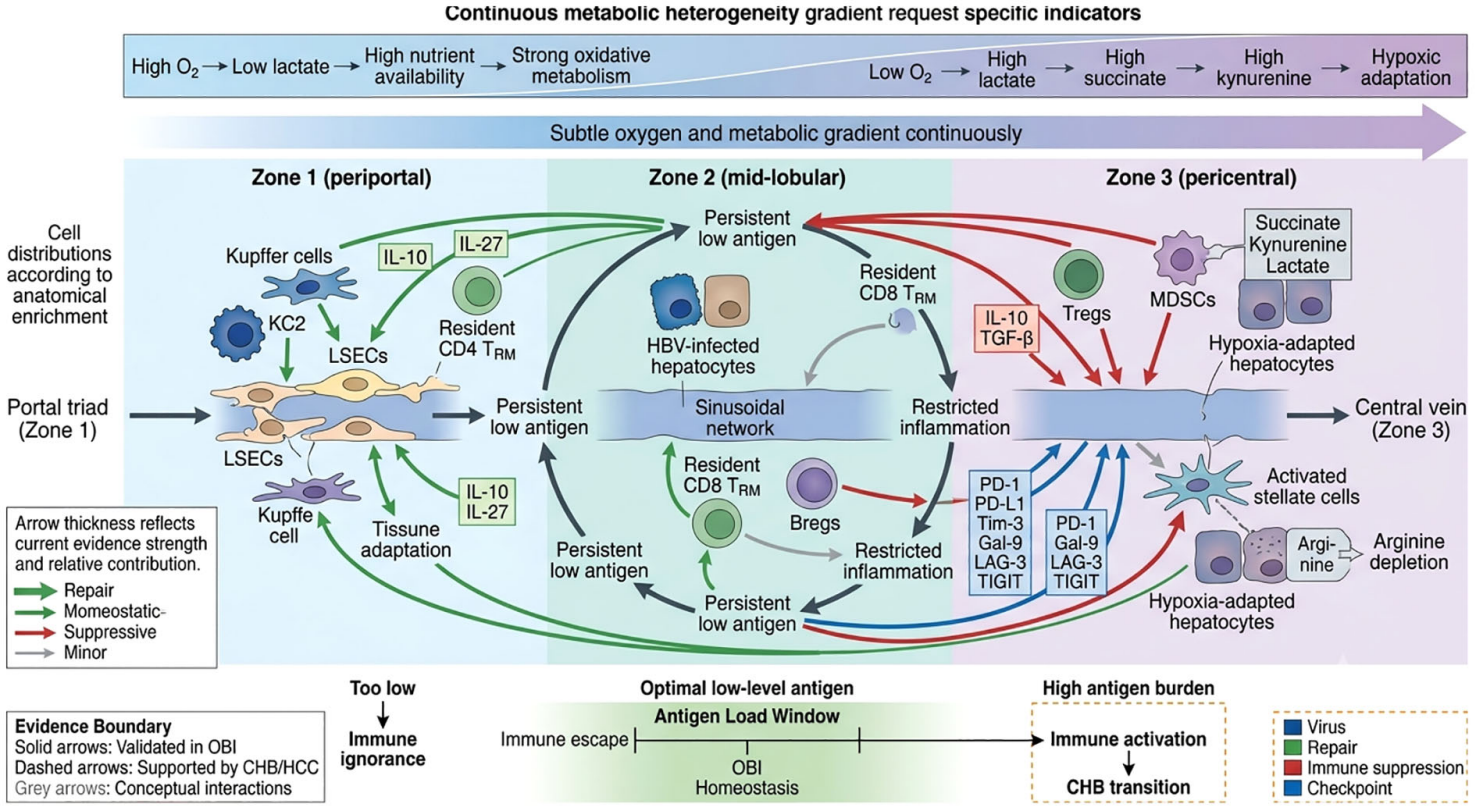
Spatial and metabolic organization of the OBI immune-mediated microcosm. The figure illustrates a spatially organized model of the occult hepatitis B virus infection (OBI) immune-mediated microcosm within the hepatic lobule. The lobular architecture is divided into periportal (Zone 1), mid-lobular (Zone 2), and pericentral (Zone 3) regions to depict anatomical heterogeneity in oxygen availability, metabolite distribution, and immune-cell localization.A continuous metabolic gradient is shown from oxygen-rich, nutrient-supported periportal regions toward hypoxic pericentral areas characterized by increased lactate, succinate, and kynurenine accumulation. Major cellular components, including Kupffer cells, liver sinusoidal endothelial cells, hepatocytes, tissue-resident memory T cells, regulatory T cells, regulatory B cells, and myeloid-derived suppressor cells, are positioned according to their reported or proposed spatial enrichment patterns. Colored arrows indicate functional interactions within the proposed microcosm: green arrows represent tissue repair and homeostatic maintenance pathways, red arrows indicate immunosuppressive mechanisms, and blue arrows denote immune checkpoint signaling. Arrow thickness reflects the relative strength of available evidence rather than quantitative interaction magnitude. Evidence boundaries are indicated by solid arrows for mechanisms supported directly by OBI or low-replicative HBV studies, dashed arrows for mechanisms inferred from chronic hepatitis B or hepatocellular carcinoma models, and gray arrows for conceptual interactions requiring further validation. The antigen-load window illustrates the proposed relationship between residual viral antigen abundance and immune behavior. Antigen levels below the maintenance range may favor immune silence, whereas an intermediate antigen range is proposed to support OBI homeostasis through calibrated immune restraint. Excessive antigen burden may exceed immune control capacity and promote transition toward chronic hepatitis B. This framework represents a hypothesis-generating model integrating current evidence and does not constitute a quantitative measurement of intrahepatic immune dynamics. A proposed operational framework for future quantitative validation is presented in **Table 3**.

The homeostatic model proposed here (**Figure 3**, **Table 2**) should be interpreted within the limits of currently available evidence. Key mechanisms, including KC2-mediated T-cell rescue, are supported primarily by HBV transgenic and human liver chimeric mouse models rather than human OBI tissue (63, 64). Likewise, existing single-cell atlases derive predominantly from patients with active chronic HBV (42, 78), whereas immunometabolic signatures in OBI have been characterized mainly in peripheral blood (71). Whether these observations accurately reflect the low-antigen intrahepatic environment of human OBI remains uncertain.

**Table 2:** Comparative immune microcosm characteristics: OBI versus representative phases of chronic HBV infection.

Feature dimension	OBI	Inactive HBsAg carrier (IC)	Chronic active hepatitis B (CAH)	References
Viral Load	Low or intermittent (typically <200 IU/mL)	Low (typically <2,000 IU/mL; often <200 IU/mL)	Persistently high (typically >2,000 IU/mL)	(2, 6)
ALT Level	Normal or mildly elevated	Normal	Persistent or fluctuating elevation	(83)
HBsAg	Negative	Positive (usually low levels)	Positive	(1, 3)
Intrahepatic cccDNA	Very low copy number, CpG hypermethylated, enriched for repressive histone marks (H3K9me3, H3K27me3)	Low copy number, partial methylation; overall reduced transcriptional activity	High copy number, active chromatin state	(32, 33, 35)
Dominant T-cell Phenotype	TRM cells, low-level activation; functional plasticity preserved	Mixed TRM and circulating memory; mild functional impairment	Exhausted T cells (PD-1hiTOXhi); deep functional defects	(59, 65, 77, 84)
Cytokine Profile	IL-10, TGF-β relative dominance	IL-10 mildly elevated; IFN-γ, TNF-α preserved at low levels	IFN-γ, TNF-α predominant, with IL-10 elevation	(67, 68, 76)
Immune Checkpoints	PD-1+CD8+ T-cell accumulation; constitutive co-inhibitory molecule expression	Low-level PD-1 expression; minimal Tim-3 upregulation	TIM-3, PD-1 highly upregulated; correlates with liver injury	(58, 73)
Metabolic Fingerprint	Low glycolysis, high kynurenine, arginine depletion	Metabolic profile comparable to healthy controls; mild tryptophan pathway activation	Enhanced aerobic glycolysis (Warburg effect); elevated kynurenine	(71, 72, 75)
Liver Fibrosis Progression	Slow; HCC can arise without cirrhosis	Slow; minimal fibrosis progression in most carriers	Rapid; cirrhosis-HCC typical pathway	(60, 61)

OBI, occult hepatitis B virus infection; IC, inactive HBsAg carrier (HBeAg-negative chronic HBV infection with persistently normal ALT and low viremia); CAH, chronic active hepatitis B (encompassing HBeAg-positive and HBeAg-negative immune-active phases with elevated ALT and/or significant histological activity). This comparison acknowledges the recognized heterogeneity of chronic HBV infection; the selected phases represent broad clinical categories, and transitional states exist. TRM, tissue-resident memory T cell; PD-1, programmed cell death protein 1; TOX, thymocyte selection-associated high mobility group box protein; TGF-β, transforming growth factor beta; IFN-γ, interferon gamma; TNF-α, tumor necrosis factor alpha; Tim-3, T-cell immunoglobulin and mucin domain-containing protein 3. “Low-level activation” indicates preserved functional potential with restrained effector responses.

**Table 3:** Proposed operational framework for future validation of the OBI immune-mediated microcosm.

Domain	Candidate parameter	Current evidence in OBI	Potential role
Residual viral activity	Serum HBV DNA	Moderate	Estimate residual viral burden
	Intrahepatic cccDNA copy number	Limited	Assess viral persistence
	HBV pgRNA	Emerging	Reflect transcriptional activity
	Quantitative HBsAg (when detectable)	Limited	Estimate antigen burden
Immune-cell balance	CD8^+^ TRM/Treg ratio	Limited	Homeostatic immune surveillance
	Kupffer-cell subsets (KC1/KC2)	Emerging	Tissue immune regulation
	MDSC frequency	Emerging	Immunosuppressive activity
Immune checkpoint	PD-1/PD-L1	Moderate	Adaptive immune restraint
	TIGIT, Tim-3, LAG-3	Emerging	Regulatory network
Soluble mediators	IL-10	Moderate	Immune tolerance
	TGF-β	Moderate	Regulatory signaling
	IFN-γ	Moderate	Antiviral immunity
	CXCL13/IL-21	Emerging	B-cell and Tfh activity
Immunometabolism	Succinate	Emerging	Inflammatory metabolism
	Lactate	Emerging	Metabolic adaptation
	Kynurenine	Emerging	Immune suppression
	Arginine availability	Emerging	T-cell functional fitness
Systems integration	Spatial transcriptomics	Experimental	Spatial immune architecture
	Multiplex imaging	Experimental	Cell–cell interaction mapping
	Longitudinal multi-omics	Future direction	Identification of homeostatic windows

No validated quantitative thresholds currently exist for defining the immune-mediated microcosm in OBI. The candidate parameters listed here are proposed as components of a standardized multidimensional framework for future prospective validation rather than established diagnostic criteria.

Spatial heterogeneity represents an additional limitation. Oxygen tension, metabolite availability, and immune cell composition vary substantially across hepatic lobular zones, yet current single-cell datasets lack sufficient spatial resolution to determine whether the proposed regulatory network operates uniformly throughout the liver. Addressing this question will require integrated spatial transcriptomic and longitudinal studies.

A further conceptual limitation concerns the proposed antigen-threshold model. Armed coexistence is hypothesized to require antigen levels sufficient to maintain tissue-resident immune surveillance while remaining below the threshold for pathogenic T-cell activation. The molecular determinants of these thresholds remain unknown but are likely to involve hepatocyte cccDNA burden, peptide–MHC density, and the local balance between costimulatory and coinhibitory signaling. Defining these parameters represents an important priority for future quantitative studies. Although this framework provides a coherent interpretation of current evidence, alternative explanations remain equally plausible.

### 3.6 The low-antigen microenvironment as a determinant of immune behavior

A defining characteristic of OBI is its exceptionally low intrahepatic antigen burden. Unlike chronic hepatitis B (CHB), where sustained viral antigen exposure drives continuous immune activation and progressive T-cell dysfunction, OBI is maintained under conditions of minimal viral antigen expression. Rather than representing a passive consequence of viral suppression, this low-antigen state may actively shape intrahepatic immune behavior, although direct evidence in human OBI remains limited.

Low antigen availability may preferentially support long-lived tissue-resident memory T (TRM) cells, which are maintained primarily by local cytokine signals rather than continuous antigen stimulation (65). Under these conditions, TRM cells may provide rapid local immune surveillance during sporadic viral transcription while minimizing systemic immune activation, thereby contributing to the maintenance of armed coexistence.

Checkpoint signaling may likewise be influenced by antigen density. PD-1 expression is induced by repeated T-cell receptor stimulation; therefore, the limited antigen exposure characteristic of OBI may permit sufficient checkpoint activity to restrain excessive immune responses without inducing the profound dysfunction observed in CHB. Consistent with this possibility, PD-1^+^CD8^+^ T cells accumulate in low-replicative HBV infection (58), yet their functional impairment may remain at least partially reversible (79).

Regulatory immune populations may also behave differently under low-antigen conditions. Whereas Treg activity depends in part on antigen-driven T-cell receptor signaling, myeloid-derived suppressor cells (MDSCs) and regulatory B cells (Bregs) are influenced predominantly by cytokine and metabolic cues. Consequently, antigen-independent suppressive mechanisms, including arginine depletion and activation of the tryptophan–kynurenine pathway, may contribute proportionally more to immune homeostasis in OBI than in high-antigen CHB (66, 72).

Direct experimental evidence supporting these mechanisms in human OBI remains limited. Most available data derive from CHB cohorts or experimental models of persistent viral infection. Future studies combining highly sensitive quantification of intrahepatic viral antigen with spatially resolved immune profiling will be essential to determine how antigen density regulates immune behavior within the OBI liver.

### 3.7 Toward an operational framework for investigating the OBI immune-mediated microcosm

The immune-mediated microcosm proposed in this Review provides a conceptual framework for understanding immune equilibrium in occult hepatitis B infection, but no validated quantitative criteria currently define this state. Existing knowledge derives largely from cross-sectional studies, low-replicative HBV models, and mechanistic investigations in chronic hepatitis B or hepatocellular carcinoma, highlighting the need for prospective validation in well-characterized OBI cohorts (41, 59, 63, 64).

Rather than relying on a single biomarker, future operationalization of this framework will likely require multidimensional integration of virological, immunological, and immunometabolic parameters. Candidate virological indicators include intrahepatic cccDNA copy number, pgRNA transcriptional activity, and serum HBcrAg levels, which collectively reflect the transcriptional competence of the residual viral reservoir (33, 80, 81). Immune-cell composition, particularly the balance among tissue-resident memory CD8^+^ T cells, Kupffer cell subsets, regulatory T cells, and myeloid-derived suppressor cells, may provide quantitative measures of immune surveillance and local immunoregulation (63, 65–67). Functional immune status may be further assessed by checkpoint molecules, including PD-1, PD-L1, TIGIT, Tim-3, and LAG-3, together with soluble mediators such as IL-10 and TGF-β (58, 59, 68, 73). Emerging immunometabolic indicators, including kynurenine, succinate, lactate, and arginine availability, may provide complementary information regarding immune adaptation within the hepatic microenvironment (52, 71, 72).

The objective is not to establish universal numerical thresholds but to develop standardized multidimensional parameter sets that can be evaluated prospectively across independent OBI cohorts. Integration of longitudinal sampling with spatial transcriptomics, multiplex imaging, and single-cell multi-omics technologies may ultimately identify quantitative homeostatic windows and transition thresholds associated with immune equilibrium, destabilization, viral reactivation, or hepatocarcinogenesis (42, 78, 82).

At present, the immune-mediated microcosm should be regarded as an operational research framework rather than a definitive biological model of OBI. Its principal value lies in generating testable hypotheses, guiding biomarker discovery, and providing a foundation for future quantitative validation across independent clinical cohorts.

## 4 Disruption of the dynamic equilibrium: from occult infection to disease progression

The immune-mediated microcosm of OBI is metastable and highly susceptible to perturbation by exogenous immunosuppression, endogenous immunosenescence, or emergent viral escape mutants. When these forces disrupt the homeostatic equilibrium described in Section 3, clinical outcomes diverge into two broad trajectories: viral reactivation presenting as clinical hepatitis, or low-grade chronic inflammation that drives fibrosis and hepatocellular carcinoma. Notably, much of the evidence supporting these trajectories derives from retrospective studies and prospective cohorts of immunosuppressed patients; whether identical dynamics operate over decades in untreated OBI carriers remains less well documented.

### 4.1 Exogenous immunosuppression and the reactivation cascade

**Figure 4:**
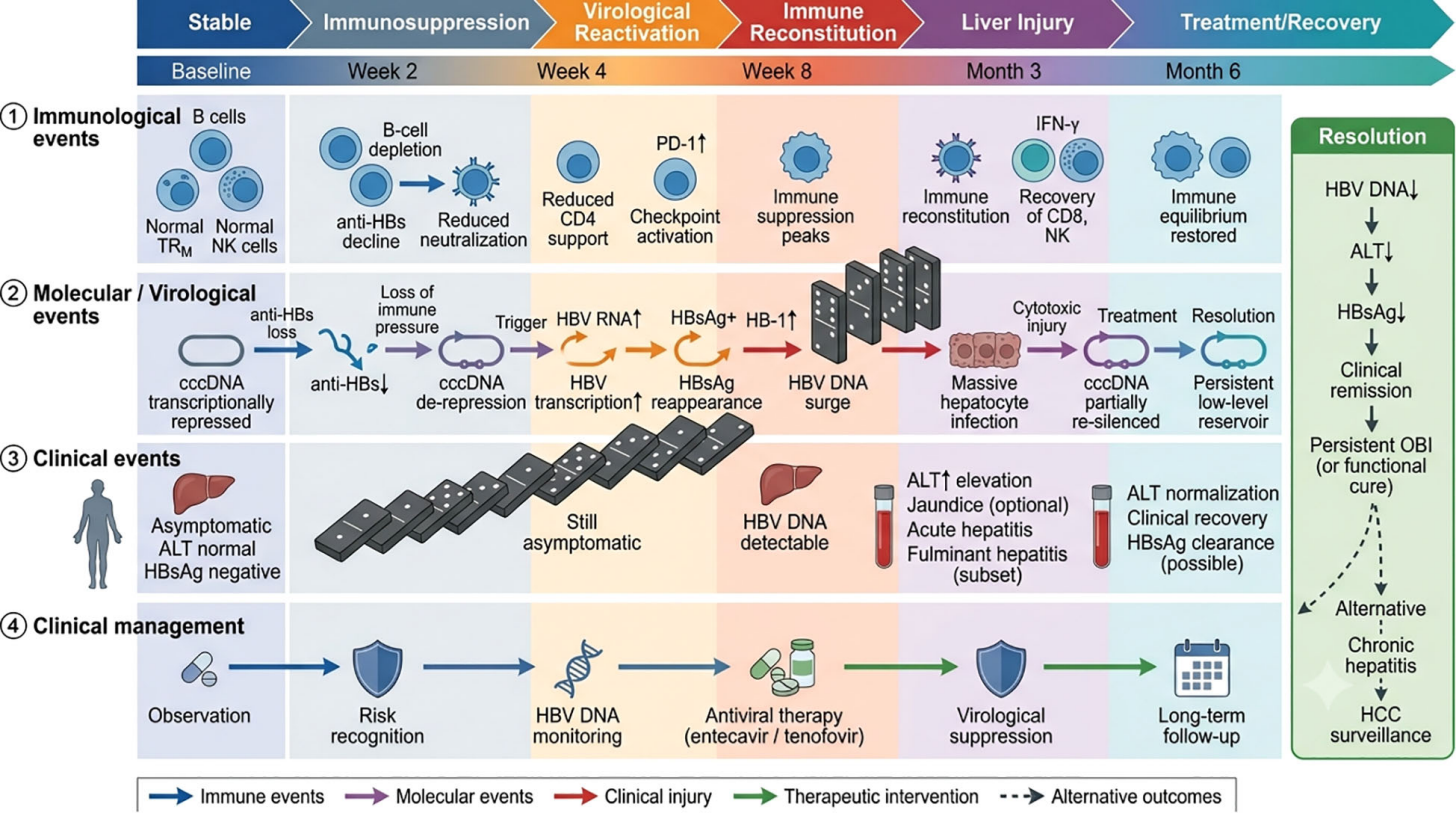
Immunopathological trajectory of OBI reactivation following immunosuppression. The figure illustrates a proposed longitudinal trajectory of hepatitis B virus (HBV) reactivation in occult HBV infection (OBI) after immunosuppression over a timescale of weeks to months. Four parallel tracks summarize immunological events, molecular and virological changes, clinical manifestations, and clinical management. Initial loss of immune control is characterized by B-cell depletion, declining anti-HBs levels, and progressive cccDNA de-repression, followed by increased HBV transcription, HBsAg reappearance, and HBV DNA expansion. Subsequent immune reconstitution may result in hepatocyte-directed immune injury, leading to ALT elevation and hepatitis flare in a subset of patients. The extended timeline incorporates antiviral intervention, virological suppression, and potential restoration of immune equilibrium, while also illustrating alternative trajectories including persistent low-level HBV reservoir and long-term HCC surveillance. The sequence and temporal intervals shown represent a conceptual synthesis of current evidence and should not be interpreted as fixed clinical timelines.

The best-established trigger of OBI reactivation is B-cell depletion induced by anti-CD20 monoclonal antibodies. Rituximab disrupts humoral immune surveillance, substantially increasing the risk of viral reactivation in anti-HBc-positive individuals (9). More potent B-cell depletion with obinutuzumab is associated with an even higher reactivation risk (10). In a prospective study of HBsAg-negative/anti-HBc-positive patients with lymphoma receiving rituximab-containing chemotherapy, the cumulative 2-year reactivation rate reached 41.5% (9). Reactivation typically follows a characteristic sequence: declining anti-HBs titers, renewed HBV replication, and, after immune recovery, immune-mediated hepatitis caused by T- and NK-cell recognition of newly infected hepatocytes (85) (**Figure 4**).

Beyond anti-CD20 therapy, OBI reactivation has been reported during conventional chemotherapy, proteasome inhibitor-based treatment, and solid organ transplantation (86–88). In liver transplantation, antiviral prophylaxis with hepatitis B immune globulin and nucleos(t)ide analogues, or selected nucleos(t)ide analog monotherapy, substantially reduces this risk (89, 90).

The introduction of direct-acting antivirals (DAAs) for hepatitis C unexpectedly highlighted the importance of host immune equilibrium in OBI. Rapid HCV clearance is thought to alter intrahepatic interferon signaling, permitting renewed HBV replication before antiviral immune control is re-established. Clinical studies, pharmacovigilance analyses, and meta-analyses have confirmed that HBV reactivation can occur during DAA therapy, although the absolute incidence remains relatively low (91–94).

Immune checkpoint inhibitors (ICIs) have emerged as another setting in which disruption of immune homeostasis may precipitate HBV reactivation. Cases have been reported across multiple malignancies, including hepatocellular carcinoma and non-hepatic solid tumors receiving PD-1 blockade (95–97). Nevertheless, current evidence remains largely observational, and prospective studies specifically evaluating reactivation risk in well-characterized OBI cohorts are still lacking.

### 4.2 Immunosenescence and viral escape

In the absence of exogenous immunosuppression, OBI homeostasis may gradually deteriorate through age-related immune remodeling and progressive loss of antiviral immune competence. Persistent low-level antigen exposure contributes to cumulative T-cell dysfunction (98). Although immune checkpoint blockade can partially restore antiviral immunity (99), recovery remains incomplete and varies according to epitope specificity. Envelope-specific T cells generally exhibit more profound dysfunction than core- or polymerase-specific populations, which often retain greater functional capacity (100). Moreover, compensatory upregulation of alternative inhibitory pathways, including Tim-3 and CTLA-4, may limit the durability of checkpoint-directed immunotherapy (99, 101).

In parallel, sustained immune selection promotes the emergence of viral escape variants. Among patients with HBV reactivation, 75.9% harbor fixed HBsAg escape mutations, with a median intra-host prevalence of 73.3% (19). PreS/S variants have also been associated with OBI reactivation in hemodialysis cohorts (102), whereas envelope hyperglycosylation may preserve HBsAg negativity despite renewed viral replication (24). Together, progressive immune dysfunction and viral adaptation may destabilize the equilibrium characteristic of OBI, favoring persistent low-level viral activity and progressive liver injury. Whether this transition occurs gradually or through discrete biological tipping points remains to be established.

### 4.3 HBV DNA integration and the path to HCC

HBV DNA integration represents one of the principal genomic events linking occult HBV infection to hepatocarcinogenesis (103). OBI is frequently detected in cryptogenic hepatocellular carcinoma, and integrated HBV DNA is present in a substantial proportion of HBsAg-negative tumors, many of which arise in the absence of cirrhosis (12). Recurrent integration near oncogenic loci, including TERT, MLL4, and CCNE1, together with long-range chromatin interactions driven by integrated viral sequences, supports a direct contribution of integration to hepatocellular transformation (104, 105). Integration-bearing hepatocyte clones may emerge early during chronic infection and undergo progressive clonal expansion throughout disease evolution (60, 106).

However, integration alone is unlikely to be sufficient for malignant transformation. The long-term persistence of integration-bearing clones without progression to cancer suggests that tumor development requires additional events, including somatic mutations, epigenetic remodeling, and progressive alterations of the hepatic immune microcosm. Whether genotype-specific integration patterns translate into differential oncogenic risk also remains uncertain (104).

Integrated viral sequences may further promote carcinogenesis through sustained HBx expression. HBx disrupts the Smc5/6 restriction complex via the DDB1–CUL4 ubiquitin ligase pathway (40) and remodels host chromatin through mechanisms that include altered histone lactylation (55). Whether continued HBx expression contributes more to hepatocarcinogenesis than the integration event itself remains unresolved. Likewise, epigenetic remodeling of residual cccDNA during progression from OBI to HCC has not been established directly in human OBI and is inferred primarily from chronic HBV and hepatocellular carcinoma models (32–35).

Building on the immune framework proposed in Section 3, HBV integration may facilitate immune escape through at least two non-exclusive mechanisms. First, expression of truncated viral proteins may generate altered antigenic repertoires that provoke localized immune activation without effective viral clearance, thereby creating a pro-mutagenic inflammatory microenvironment. Second, integration-associated chromatin remodeling may impair antigen presentation, reducing immune recognition and permitting silent clonal expansion (104, 105). The relative contribution of these mechanisms is likely to vary according to the genomic context of individual integration events and the surrounding immune microenvironment. Both ultimately favor the emergence of hepatocyte clones that escape long-term immune control and progress toward hepatocellular carcinoma.

### 4.4 Immune microcosm reprogramming during hepatocarcinogenesis

Persistent low-grade inflammation may progressively reprogram the OBI immune-mediated microcosm from a state that constrains viral replication toward one that permits tumor development. Expansion of MDSCs, Treg cells, and IL-10-producing Kupffer cells, together with activation of the tryptophan–kynurenine pathway, establishes an immunoregulatory environment that suppresses antiviral effector function while facilitating persistence of integration-bearing hepatocyte clones (66, 67, 72, 76). Similar immune landscapes have been described in HCC, where MDSC accumulation correlates with advanced disease and poor prognosis (107). Whether identical mechanisms operate during the transition from OBI to HCC remains uncertain.

An alternative interpretation should also be considered. Integrated HBV DNA detected in HCC may, in some tumors, represent a molecular remnant of previous infection rather than an active oncogenic driver. This interpretation is consistent with observations that integration-bearing hepatocyte clones can persist for decades without malignant transformation (60). Under this model, the immune remodeling described above, which includes expansion of suppressive immune populations and metabolic adaptation, may reflect age-related immune remodeling and chronic low-grade inflammation rather than a direct causal pathway from OBI to HCC. Consequently, therapeutic elimination of integration-bearing clones alone may not necessarily reduce HCC risk.

**Figure 5:**
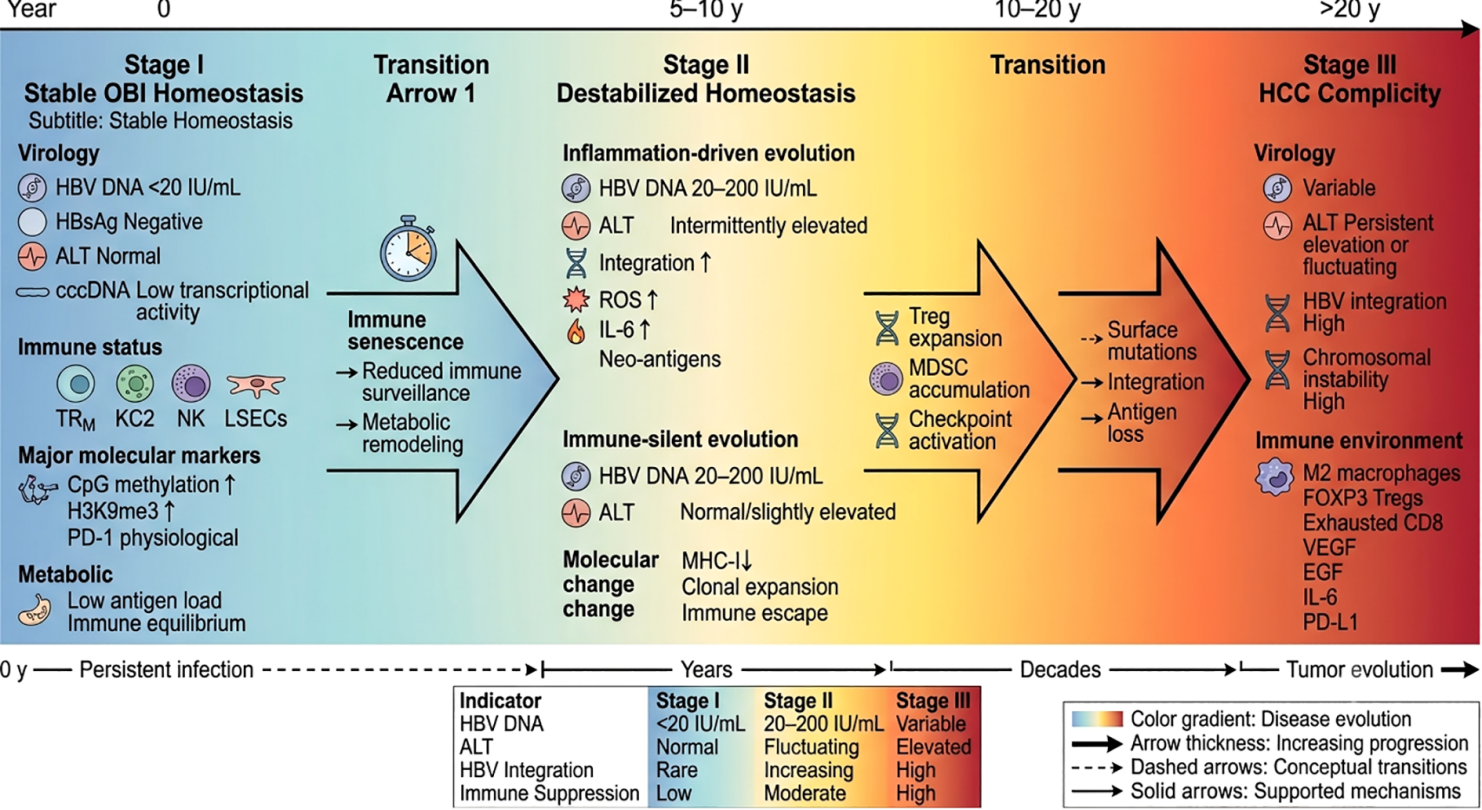
Evolutionary trajectory from occult HBV infection homeostasis to HCC-associated clonal evolution. The figure presents a conceptual model describing the progressive destabilization of occult HBV infection (OBI) homeostasis over years to decades. Rather than representing discrete disease stages, the framework illustrates a continuum from stable immune control to altered host–virus equilibrium and eventual hepatocellular carcinoma (HCC)-associated evolution. Stage I represents stable OBI homeostasis, characterized by low-level viral persistence, transcriptionally restricted cccDNA, preserved immune surveillance, and a regulatory hepatic environment. Transition toward Stage II is associated with progressive immune aging, metabolic remodeling, and reduced surveillance capacity. Stage II represents a destabilized intermediate state in which viral adaptation, HBV DNA integration, and immune remodeling generate heterogeneous evolutionary trajectories. Two conceptual routes are illustrated: an inflammation-associated pathway involving increased antigenic stimulation and focal immune activation, and an immune-silent pathway characterized by reduced antigen presentation and clonal expansion under limited immune recognition. Stage III depicts HCC-associated evolution, characterized by increased HBV integration burden, genomic instability, immune remodeling, and enrichment of tumor-supportive immune features, including M2 macrophages, regulatory T cells, exhausted CD8^+^ T cells, and pro-tumorigenic mediators. Representative indicators, including HBV DNA levels, ALT activity, integration burden, and immune suppression status, are provided to illustrate relative changes across the proposed trajectory. Color gradients indicate progressive deviation from immune homeostasis rather than inflammatory intensity. Solid arrows represent biologically supported relationships, whereas dashed arrows indicate conceptual transitions requiring further validation. The temporal intervals and evolutionary transitions shown are hypothesis-generating and should not be interpreted as fixed clinical timelines.

The evolutionary model summarized in **Figure 5** should therefore be regarded as a conceptual framework rather than a validated disease trajectory. Stage I is supported mainly by cross-sectional immunophenotyping studies (60, 106), Stage II by longitudinal reactivation cohorts (9), and Stage III by analyses of established HCC specimens (12, 103, 104, 107). Direct longitudinal evidence linking these stages remains limited, and the timing, transition thresholds, and proportion of OBI carriers who ultimately develop HCC remain unknown. Prospective studies integrating spatial transcriptomics, longitudinal sampling, and single-cell multi-omics will be required to determine whether the proposed trajectory accurately reflects human disease progression.

## 5 From mechanism to clinical translation

The preceding chapters examine the clinical trajectory of OBI as governed by a metastable equilibrium among viral quasispecies, host epigenetic constraints, and the intrahepatic immune microcosm. At this juncture, a pivotal question is whether understanding these interactions can realistically inform clinical interventions to improve patient outcomes. Equally critical is whether healthcare systems in highly endemic regions can support these resource-intensive strategies. Currently, the evidence to definitively answer both questions remains incomplete.

### 5.1 Refining diagnosis: beyond HBsAg negativity

Ultrasensitive HBsAg assays are narrowing the detection gap left by conventional serology. Chemiluminescent platforms with detection limits near 0.005 IU/mL reclassify samples previously deemed HBsAg-negative, uncovering mutation spectra responsible for viral escape across various genotypes (4, 51). Similarly, the iTACT-HBsAg assay detects residual HBsAg in patients classified as seronegative by conventional methods (108), a finding supported by longitudinal cohort studies of patients with documented HBsAg seroclearance (109). At the molecular level, CRISPR-Cas13a coupled with recombinase polymerase amplification achieves single-copy HBV DNA detection within 30 minutes (110).

However, comprehensively assessing the intrahepatic microcosm requires parameters beyond mere viral detection. Serum HBcrAg levels directly correlate with intrahepatic cccDNA transcriptional activity (80), while serum HBV RNA and pgRNA levels reflect cccDNA transcription, offering insights into the depth of viral latency (81, 111). A combined diagnostic pathway incorporating HBcrAg, ultrasensitive HBV DNA, and pgRNA achieved 78.9% sensitivity and 100% specificity, with HBcrAg positivity strongly correlating with elevated HCC risk (112). Furthermore, OBI status correlates with increased liver fibrosis severity (113). To this end, a non-invasive serum panel based on CK18-M65, CK18-M30, and GP73 detects occult fibrosis in HBV-infected individuals with normal ALT levels, yielding an area under the curve (AUC) of 0.942 (114). Nonetheless, a key limitation is that these biomarker panels have been validated predominantly in cross-sectional cohorts. Whether dynamic changes in biomarker trajectories can predict clinical decompensation remains untested, and their long-term capacity to forecast individual-level outcomes over years of follow-up has yet to be established.

### 5.2 Risk stratification and precision prophylaxis

Accurately identifying OBI carriers at high risk for reactivation is a prerequisite for targeted prophylaxis. A decision curve analysis of 8,034 patients demonstrated that prophylactic antiviral therapy offers a net benefit across various immunosuppressive regimens, with a number needed to treat (NNT) as low as 8 for rituximab-based protocols (115). Although host HLA variations associate with OBI susceptibility at the population level (14, 15), a multicenter whole-exome study found that a standard clinical model effectively differentiated OBI from overt carriers, yielding AUCs of 0.898 to 0.910. This model incorporated age, HBV DNA threshold cycle (Ct) values, anti-HBs, anti-HBc, and anti-HBe (116). Notably, host genomic variants added no incremental discriminatory value to this model (116). This suggests that risk stratification can be readily achieved in the near term using widely available serological indices, bypassing the need for costly genomic sequencing. Nevertheless, a substantial gap persists between risk identification and the implementation of preemptive strategies in many endemic settings. Furthermore, whether a risk score validated in a Chinese blood donor cohort will perform reliably in sub-Saharan African populations characterized by distinct HLA landscapes and viral genotypes remains unestablished.

### 5.3 Targeting the viral life cycle

Numerous antiviral strategies targeting distinct nodes of the HBV life cycle are currently undergoing clinical evaluation. For instance, the entry inhibitor bulevirtide reduced the pool of HBV/HDV-co-infected hepatocytes in a phase III trial (117, 118), while the antisense oligonucleotide bepirovirsen achieved sustained HBsAg and HBV DNA clearance in 9% to 10% of patients in a phase IIb study (119). Additionally, small interfering RNA (siRNA) therapeutic agents, such as ARC-520 and ALN-HBV02, induce dose-dependent HBsAg reductions in patients undergoing nucleos(t)ide analogue therapy (120, 121). In preclinical models, non-cleaving CRISPR base editors successfully inactivate the core open reading frames of both integrated HBV DNA and episodic cccDNA (122), and Cas9 ribonucleoprotein complexes cleave cccDNA *in vitro* with minimal off-target effects (123). Although the toll-like receptor 7 (TLR7) agonist vesatolimod failed to reduce HBsAg levels as a monotherapy, the innate immune activation it triggers provides a rational basis for combination regimens (124). Crucially, the majority of these novel therapeutics have been evaluated in patients with active chronic hepatitis B rather than OBI carriers; whether the lower antigen burden characteristic of OBI modifies the efficacy or safety profiles of these agents remains systematically unstudied.

### 5.4 Restoring immune surveillance

Because the OBI microcosm is defined by active restraint rather than absolute immunological incompetence, reviving host immune surveillance represents a viable strategy toward achieving a functional cure. In a phase Ib/IIa trial, the therapeutic vaccine BRII-179 elicited anti-HBs seroconversion in approximately 30% of recipients and restored multifunctional HBV-specific T-cell responses (125). Concurrently, HBsAg-targeted chimeric antigen receptor (CAR)-T cells exhibit robust *in vivo* antiviral activity and deplete cccDNA reservoirs in human liver chimeric mouse models (126). Furthermore, a phase I trial of HBV-specific T-cell receptor (TCR)-T cells in patients with recurrent HBV-related HCC post-transplantation demonstrated acceptable safety along with dual antitumor and antiviral efficacy (127). Recent evidence also indicates that HBV-targeting CAR-natural killer (NK) cells may offer a highly favorable safety profile characterized by a low risk of cytokine release syndrome (128).

Alternatively, the epigenetic “shock and kill” strategy aims to pharmacologically reactivate latent cccDNA transcription, thereby exposing infected hepatocytes to host immune clearance. This approach leverages insights into SIRT3-catalyzed H3K9 deacetylation (37) and HBx-mediated antagonism of SETDB1-deposited H3K9me3 (39). Additionally, acetyl-CoA-based metabolic interventions can reprogram exhaustion-associated epigenetic marks to revive CD8+ T-cell antiviral functions (77), while modulating the histone lactylation landscape holds promise for combined metabolic-immunotherapeutic strategies in HBV-related HCC (54). These emerging strategies are summarized in **Table 4**.

**Table 4:** Potential therapeutic targets and investigational strategies across different mechanistic layers in OBI.

Mechanistic layer	Specific target/strategy	Representative agent	Development phase	Advantages	Challenges (including safety concerns)	References
Viral Entry	NTCP receptor blockade	Bulevirtide	Phase III	Blocks replenishment of the cccDNA pool	No effect on established cccDNA; long-term safety in OBI carriers not established	(117, 118)
Viral Transcription	Antisense inhibition of HBV mRNA	Bepirovirsen (ASO)	Phase IIb	Can achieve sustained HBsAg and HBV DNA clearance	Requires subcutaneous injection; limited response rate (9–10%); injection site reactions; hepatic flares reported in a subset of patients	(119)
Viral Transcription	RNA interference	ARC-520, ALN-HBV02 (siRNA)	Phase II	Deep HBsAg reduction; dose-dependent efficacy	Requires combination with immunotherapy; potential for off-target RNA silencing; hepatobiliary toxicity signals in preclinical models	(120, 121)
cccDNA Elimination	CRISPR/Cas base editing	SpRY-ABE8e/CBE4-max	Preclinical	Permanent cccDNA inactivation without double-strand breaks	Delivery: AAV vector hepatotoxicity documented in primates and clinical gene therapy trials; Immunogenicity: Pre-existing anti-Cas9 antibodies present in 58% (anti-SpCas9) to 78% (anti-SaCas9) of healthy donors; SaCas9-specific T cells detectable in 78% of donors; Off-target: Risk of chromosomal rearrangements through inadvertent editing of integrated HBV DNA fragments; long-term genotoxicity unknown	(122, 123, 130, 131)
Innate Immunity	TLR7 agonism	Vesatolimod (GS-9620)	Phase II	Activates intrahepatic ISGs; oral bioavailability	Limited monotherapy efficacy (no significant HBsAg decline); systemic immune activation may disrupt OBI immune equilibrium; flu-like symptoms common	(124)
Adaptive Immunity	Therapeutic vaccine	BRII-179 (PreS1/PreS2/S)	Phase Ib/IIa	Restores T/B cell responses; anti-HBs seroconversion in ~30% of recipients	Requires combination strategies for durable responses; injection site reactions; theoretical risk of vaccine-induced hepatic inflammation in OBI carriers	(125)
Adaptive Immunity	HBV-specific CAR-T	HBsAg-CAR T cells	Phase I	Demonstrated *in vivo* antiviral activity; reduces cccDNA levels in human liver chimeric mouse model	CRS: Risk of cytokine release syndrome, particularly upon antigen encounter in the liver; On-target/off-tumor: Potential recognition of HBsAg-expressing non-malignant hepatocytes causing hepatic injury; Manufacturing: Autologous manufacturing complexity and cost; Durability: CAR-T cell persistence and functionality in the immunosuppressive OBI milieu unproven	(126, 128)
Adaptive Immunity	HBV-specific TCR-T	mRNA electroporated TCR-T	Phase I	Dual antitumor/antiviral activity; demonstrated safety in post-transplant HCC patients	CRS: Cytokine release syndrome risk; Manufacturing: Complex personalized manufacturing (HLA restriction); limited applicability across diverse HLA haplotypes; Durability: Transient mRNA expression may limit long-term efficacy	(127)
Epigenetics	Deacetylation/demethylation	HDACi, SIRT inhibitors	Preclinical	“Shock” the latent virus; reactivates cccDNA transcription to expose infected hepatocytes	Iatrogenic hepatitis risk: Non-selective global transcriptional activation may trigger acute hepatitis indistinguishable from spontaneous reactivation; Selectivity: Differential effects on integrated HBV DNA vs. episomal cccDNA not characterized—integrated DNA reactivation may increase oncogenic HBx expression; Safety window: Narrow therapeutic index in asymptomatic OBI carriers	(9, 37, 39)
Metabolism-Immunity	Acetyl-CoA/lactylation modulation	Metabolic intervention	Preclinical	Restores exhausted T-cell function through epigenetic reprogramming	Specificity: Targeting specificity to HBV-specific exhausted T cells not yet optimized; Off-target metabolism: Systemic metabolic modulation may affect non-immune tissues; Evidence gap: Data derive from HCC and acute liver failure models (Tier 3); relevance to OBI low-replication state unproven	(36, 54–56, 66, 77)

NTCP, sodium taurocholate co-transporting polypeptide; ASO, antisense oligonucleotide; siRNA, small interfering RNA; CRISPR, clustered regularly interspaced short palindromic repeats; Cas, CRISPR-associated protein; AAV, adeno-associated virus; TLR7, Toll-like receptor 7; ISG, interferon-stimulated gene; CAR-T, chimeric antigen receptor T cell; TCR-T, T-cell receptor-engineered T cell; HDACi, histone deacetylase inhibitor; SIRT, sirtuin; CRS, cytokine release syndrome; HCC, hepatocellular carcinoma. Development phases are current as of the literature search date. Safety concerns listed in bold represent risks that are particularly salient for the OBI population, given the predominantly asymptomatic status and long-term clinical stability of OBI carriers, which demands a substantially higher safety threshold than that accepted in oncology. References for specific safety data are provided alongside the original therapeutic references; see the main text (Section 5.4) for detailed safety discussions.

However, these approaches present significant safety concerns that warrant rigorous evaluation. The “shock” component of the strategy is particularly problematic, as histone deacetylase (HDAC) inhibitors induce global transcriptional activation rather than targeting viral templates selectively (37, 39). In an OBI carrier who has maintained a stable immune equilibrium for decades, such non-specific pharmacological disruption could precipitate severe iatrogenic acute hepatitis. Given that spontaneous HBV reactivation can rapidly progress to fulminant hepatic failure (9), deliberate transcriptional induction carries an inherent risk of provoking identical clinical crises. Although a phase I trial of the HDAC inhibitor vorinostat in HIV-infected individuals on suppressive antiretroviral therapy reported acceptable tolerability (129), HIV proviruses and HBV cccDNA episomes are regulated by fundamentally distinct transcriptional mechanisms; thus, safety data from HIV latency reversal cannot be directly extrapolated to the context of OBI.

Regarding gene-editing approaches, pre-existing adaptive immunity to Cas9 proteins presents a notable hurdle. Anti-SaCas9 and anti-SpCas9 antibodies are detectable in 78% and 58% of healthy donors, respectively, while SaCas9-specific T cells are present in 78% (130). Introducing such highly immunogenic bacterial proteins into an OBI-affected liver, where immune homeostasis relies on a delicate network of regulatory checks, could destabilize local immune tolerance. Furthermore, adeno-associated virus (AAV) vector-mediated delivery introduces risks of hepatotoxicity, as high-dose systemic AAV administration has been associated with significant liver injury in both preclinical models and clinical gene therapy trials (131).

Regulatory and ethical frameworks for OBI also diverge substantially from those established in oncology. While CAR-T cell therapies for relapsed B-cell malignancies are indicated for patients with advanced disease and limited life expectancy, OBI carriers are typically asymptomatic and remain clinically stable for decades. Consequently, deploying interventions that carry risks of cytokine release syndrome, insertional mutagenesis, or long-term off-target genomic modifications in an otherwise healthy population demands a far more stringent safety threshold than that accepted in oncology—a benchmark that remains an open question.

### 5.5 Precision classification and the accessibility gap

**Figure 6:**
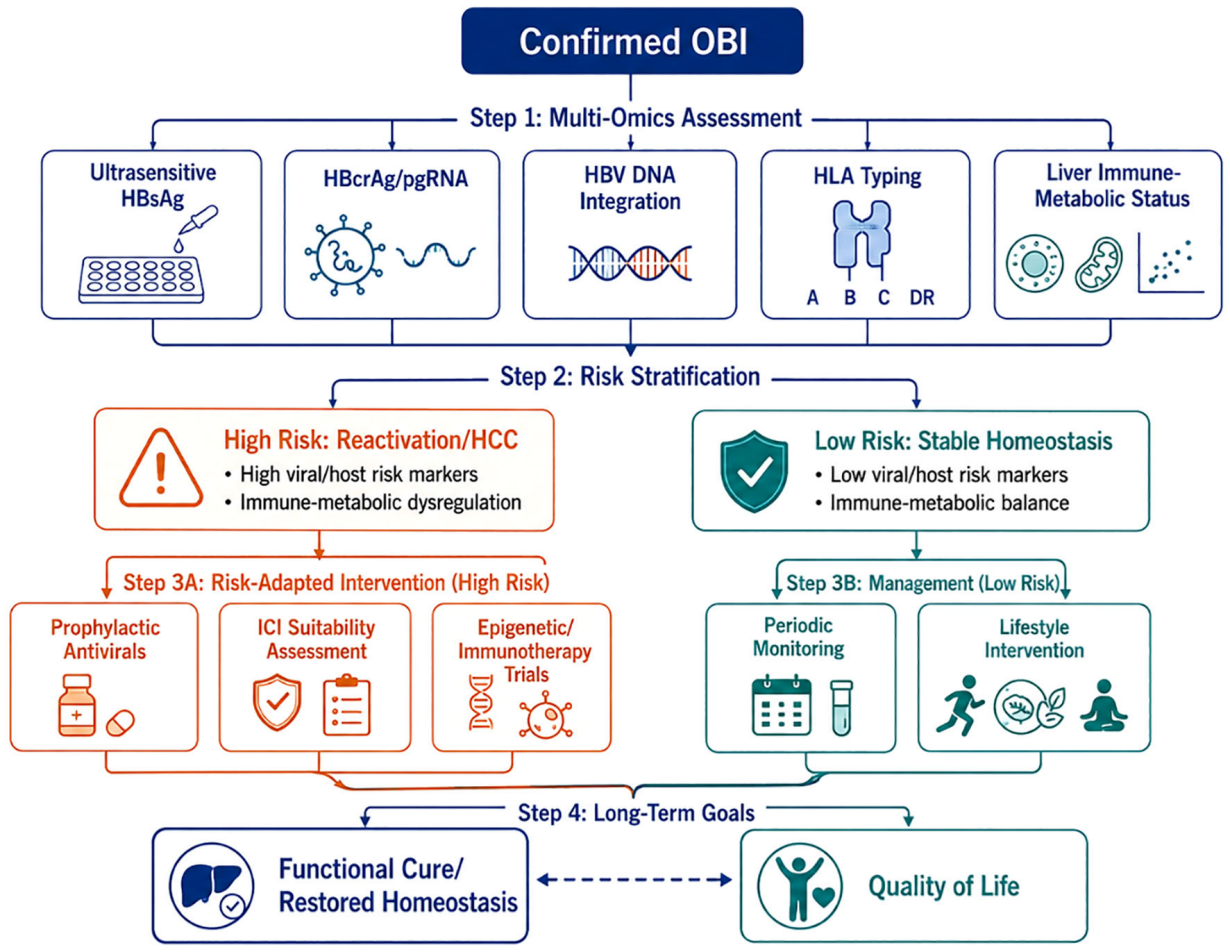
Precision medicine roadmap for OBI management: A central decision tree originates from “Confirmed OBI.” The first level is a multi-omics comprehensive assessment, including ultrasensitive HBsAg, HBcrAg, pgRNA, HBV DNA integration status, HLA typing, and liver immune-metabolic status. The second level is risk stratification, branching into “High Risk: Reactivation/HCC” and “Low Risk: Stable Homeostasis.” The high-risk pathway connects to prophylactic antiviral therapy, immune checkpoint inhibitor suitability assessment, and epigenetic/immunotherapy clinical trials; the low-risk pathway connects to periodic monitoring and lifestyle intervention. Both pathways converge at endpoints labeled “Functional Cure/Restored Homeostasis” and “Quality of Life”.

Multi-omics-based stratification of OBI is rapidly evolving. Integrated proteomic and metabolomic analyses successfully differentiate OBI from chronic HBsAg-positive infection based on unique amino acid metabolic profiles (71). Concurrently, a polygenic risk score incorporating 13 susceptibility single-nucleotide polymorphisms (SNPs), clinical HBV parameters, alcohol history, and cirrhotic status achieved a predictive AUC of 0.86 for HCC development (132). Additionally, the extent of B-cell functional rejuvenation following PD-1 blockade holds promise as a predictive biomarker for immunotherapy responsiveness (79). These diverse components can be synthesized into an integrated precision management framework (**Figure 6**).

However, this framework must be contextualized within global epidemiological realities. The global burden of OBI is heavily concentrated in low- and middle-income countries (LMICs) across sub-Saharan Africa, Southeast Asia, and the Western Pacific (6, 7)—regions that frequently lack the specialized infrastructure required for advanced cellular manufacturing, CRISPR delivery, and intensive post-treatment monitoring. Currently, autologous CAR-T products cost between $373,000 and $475,000 USD per patient in high-income jurisdictions, excluding the substantial costs associated with managing toxicities (133). While off-the-shelf allogeneic CAR-NK therapies may reduce upfront manufacturing expenses, they remain dependent on good manufacturing practice (GMP) facilities, cryopreservation infrastructure, and robust cold chains. Even if scale-up efforts lower per-patient production costs, the global pool of OBI carriers comprises tens of millions of individuals, rendering universal access unattainable under existing healthcare financing paradigms.

Consequently, the near-term application of these advanced therapeutics will likely be restricted to highly selected subgroups, such as carriers with biopsy-confirmed pre-malignant clonal expansion, patients facing imminent high-intensity immunosuppressive regimens, or individuals presenting with early-stage HCC. For the vast majority of OBI carriers, routine monitoring paired with targeted prophylactic nucleos(t)ide analog therapy will remain the clinical standard. Concurrently, the primary public health priorities must continue to focus on strengthening blood-product screening and expanding preventive vaccination coverage. The precision framework proposed herein is therefore valuable not as a blueprint for universal complex intervention, but as a risk-stratification tool to isolate the subset of patients whose risk of disease progression warrants aggressive clinical oversight. Ultimately, the vast majority of OBI carriers worldwide will neither require nor gain access to these advanced therapeutic modalities—a reality that should temper expectations regarding the immediate clinical impact of this precision framework.

## 6 Conclusions and future directions

One important limitation of the evidence reviewed here is its predominant reliance on cross-sectional studies, experimental models, and indirect mechanistic inference. Whether the dynamic processes proposed in this framework operate similarly within individual OBI carriers over decades remains unknown. Accordingly, the immune-mediated microcosm should presently be regarded as a hypothesis-generating framework rather than a definitive biological model.

This Review proposes that long-term OBI persistence is best understood as a dynamic equilibrium emerging from coordinated interactions among viral genetic adaptation, host epigenetic regulation, and intrahepatic immune homeostasis. Disruption of this equilibrium by immunosuppression, immunosenescence, or viral immune escape may shift the system toward viral reactivation or hepatocarcinogenesis. Whether similar ecological principles contribute to other persistent infections, including HIV latency or mycobacterial persistence, remains an open question requiring direct investigation.

Several priorities should guide future research. First, prospective longitudinal studies integrating spatial transcriptomics, single-cell multi-omics, and quantitative virological profiling are needed to validate the proposed immune-mediated microcosm and to identify operational biomarkers of immune equilibrium (42, 78, 82). Second, non-invasive monitoring strategies combining serum HBcrAg, pgRNA, ultrasensitive HBsAg, and fibrosis-associated biomarkers warrant evaluation for longitudinal risk stratification, although their predictive performance remains to be established (71, 80, 81, 113, 114). Third, therapeutic development should focus not only on suppressing residual viral reservoirs but also on restoring immune homeostasis. Whether antiviral platforms, including base editing, antisense oligonucleotides, and CRISPR-based approaches, can be safely combined with immune or metabolic modulation requires careful clinical evaluation (119–123).

Safety and implementation remain equally important considerations. Gene-editing technologies, epigenetic modulators, and immune-based therapies face substantial challenges, including off-target effects, vector-associated hepatotoxicity, and immunogenicity (9, 130, 131, 133). Moreover, because the global burden of OBI is concentrated in resource-limited settings, risk stratification, optimized surveillance, and affordable antiviral prophylaxis are likely to remain the principal clinical strategies for most patients, whereas advanced molecular therapies may initially be applicable only to carefully selected high-risk populations.

The broader public health implications likewise deserve continued attention. Persistent transfusion-transmitted HBV in highly endemic regions highlights the need for improved ultrasensitive screening strategies and region-specific implementation within existing hepatitis elimination programs (3, 4, 134).

Overall, the principal contribution of this Review is not the establishment of a new biological paradigm, but the proposal of a structured framework for investigating long-term immune equilibrium in OBI. By integrating viral evolution, host regulation, and intrahepatic immune ecology into a unified conceptual model, this framework generates experimentally testable hypotheses, highlights priorities for biomarker development, and provides a foundation for future prospective studies. **Table 5** summarizes the major unresolved questions that must be addressed before this framework can be translated into precision risk assessment and clinical management.

**Table 5:** Key challenges and unresolved questions in OBI research.

Research area	Key challenges and unresolved questions	Impact on the “Immune-Mediated Microcosm” Framework
Virology	Is cccDNA transcription in OBI “completely silenced” or subject to “low-level leaky transcription”? Current detection methods (e.g., ddPCR) have limited sensitivity in the low-copy setting, and liver biopsy introduces spatial sampling bias.	Determines whether the virus maintains a basal level of antigenic stimulation essential for sustaining the poised state of tissue-resident memory T cells. Complete silencing would imply that immunological memory is maintained in a near-antigen-free environment.
Immunology	1. What are the precise antigen-density thresholds that define the homeostatic set-point of the immune-mediated microcosm? 2. Does a quantifiable effector-to-regulatory cell ratio (e.g., TRM/Treg) exist at which equilibrium is disrupted? 3. To what extent do local concentrations of immunosuppressive cytokines (IL-10, TGF-β) and metabolites (kynurenine) determine the state of immune hyporesponsiveness?	The absence of these quantitative parameters represents the major operational weakness of the current conceptual framework. Precisely defining these thresholds is a prerequisite for model validation and the development of objective risk-stratification tools.
Mechanisms of Carcinogenesis	1. What are the decisive co-factors (e.g., somatic mutations, telomere attrition) that drive the transition of an integration-bearing clone from benign expansion to malignant transformation? 2. How can we prospectively distinguish whether detected HBV DNA integration is a functional oncogenic driver or simply a molecular scar of past infection?	Clarifying these two issues will determine whether “immune complicity” is an active, targetable carcinogenic process or a passive epiphenomenon of age-related immune senescence. This distinction fundamentally shapes the design of therapeutic interventions.
Clinical Translation	1. How can a non-invasive “liquid biopsy” panel be developed to dynamically monitor the immune microcosm for homeostatic shifts, rather than merely tracking viral load? 2. For asymptomatic OBI carriers, how should the potential benefit of high-risk interventions (e.g., “shock and kill”) be weighed against the risk of iatrogenic injury?	These two translational bottlenecks represent the primary barriers to moving the precision medicine roadmap from a conceptual framework into clinical practice.
Global Public Health	In HBV-hyperendemic LMICs, how can costly multi-omics technologies and advanced therapies be translated into affordable, accessible, and implementable risk-stratification protocols?	This is the ultimate test of whether the framework can generate real-world clinical impact, requiring tiered management strategies tailored to local healthcare infrastructure.
Theoretical Framework	How can the proposed ecological network properties of the immune-mediated microcosm—specifically functional redundancy and critical transitions—be experimentally validated and mathematically modeled using systems biology approaches?	This step is essential for advancing the framework from a heuristic metaphor to a rigorously testable scientific theory.
